# An efficient component of the redundancy calibration program to ensure equipment stability by assaying HDR Ir‐192 sources at the time of replacement

**DOI:** 10.1002/acm2.14509

**Published:** 2024-09-05

**Authors:** Javier Vijande, Vicente Carmona, Françoise Lliso, Facundo Ballester, Jose Perez‐Calatayud

**Affiliations:** ^1^ Departamento de Física Atómica Molecular y Nuclear Universitat de Valencia (UV) Burjassot Spain; ^2^ Unidad Mixta de Investigación en Radiofísica e Instrumentación Nuclear en Medicina (IRIMED), Instituto de Investigación Sanitaria La Fe (IIS‐La Fe) Universitat de Valencia (UV) València Spain; ^3^ Instituto de Física Corpuscular IFIC (UV‐CSIC) Burjassot Spain; ^4^ Radiotherapy Department, Hospital Universitari i Politècnic La Fe de València València Spain; ^5^ Radiotherapy Department. Hospital Clínica Benidorm Alicante Spain

**Keywords:** brachytherapy

## Abstract

**Background:**

Brachytherapy (BT) treatments involving temporary high‐dose rate (HDR) sources are extensively employed in clinical practice. Ensuring the consistency of all measurement equipment at the hospital level is crucial, requiring a robust redundancy and consistency program. This enables the institution to verify the stability of the dosimetry system over time.

**Purpose:**

To describe, justify, and analyze a component of the redundancy program of the calibration protocols followed by the Radiotherapy Department of the Hospital Universitari i Politècnic La Fe (València, Spain) during the last 10 years for the case of HDR BT as an additional component to ensure long term stability of the measurement equipment.

**Methods:**

At the time the HDR BT source is replaced, its Air Kerma Strength (*S_K_*) is measured. By comparing this value with the one obtained at the time of installation (corrected by decay), a clear determination of the stability of the measurement equipment can be performed.

**Results:**

Difference between *S_K,vendor_* and *S_K,hosp_* as a function of the measurement date is reported for a 10 years’ period. All measurements are well within the ±3% tolerance level recommended in current international guidelines. Percentage differences of *S_K,hosp_* values at the time of replacement compared to *S_K,hosp_* ones at the time when the source was installed are within the ±0.5% range, reflecting oscillations around a null deviation.

**Conclusions:**

The method proposed allows any hospital to ensure a redundancy component of the long‐term stability of all equipment involved in BT measurements in a very simple and time efficient manner. Additionally, it enables the hospital to maintain a detailed log of historical differences, facilitating the identification and correction of potential systematic deviations over time.

## 1 INTRODUCTION

Brachytherapy (BT), a form of internal radiation therapy, involves the placement of radioactive sources in or near the tumor site. This localized delivery of radiation distinguishes BT from external beam radiation therapy, offering advantages in targeting tumors while minimizing damage to surrounding healthy tissues.

This technique is widely employed as an effective treatment approach for cervix, prostate, breast, and skin cancers, offering a minimally invasive alternative to surgery, with comparable outcomes and fewer associated complications. Its effectiveness extends to tumors situated in diverse anatomical regions such as the brain, head and neck (e.g., lips or tongue), eye, trachea and bronchi, digestive system, urinary tract (e.g., bladder, rectum, anus, urethra, or penis), female reproductive tract (including uterus, vagina, and vulva), and other soft tissues.^1^ The utilization of BT, either as a standalone treatment or in conjunction with other radiotherapeutic modalities, yields favorable outcomes. As an example, combining BT with external beam radiation elevates the 4‐year survival rate for cervical cancer patients from 51% to 64%,^2^ and its application enables the preservation of eye functionality in choroidal melanoma patients.^3^

Central to the success of BT is the precise calibration of radiation source strength and the strict adherence to standardized protocols formulated for national and international organizations.^4^, ^5^, ^6^, ^7^, ^8^ Calibration ensures accurate delivery of the prescribed radiation dose, minimizing the risk of under‐ or over‐dosage. Consistent adherence to established protocols is essential to ensure uniformity and safety across treatment centers, optimizing therapeutic outcomes, and minimizing adverse effects.

This manuscript focuses on the calibration protocols followed by the Radiotherapy Department of the Hospital Universitari i Politècnic La Fe (València, Spain) during the last 10 years for the particular case of BT treatments using temporary application of ^192^Ir high‐dose rate (HDR) sources. In the following, we describe the solution implemented in this center as an additional component of the redundancy program (protocols to verify the proper behavior of the equipment available in the center by using an additional set of sources and detectors) to guarantee the long‐term stability of the measurement equipment, that is, assaying the source to be replaced and comparing its Air Kerma Strength (*S_K_*) with the value measured when installed. This procedure has been incorporated as a recommendation within the redundancy program components in the “*GEC‐ESTRO ACROP recommendations on calibration and traceability of HE HDR‐PDR photon‐emitting brachytherapy sources at the hospital level* ^5^” but its methodology, rationale and results has not been described elsewhere in the literature, and this has been the motivation for the present work.

## 2 METHODS

High dose rate BT ^192^Ir sources are typically replaced every 3−4 months. The protocol followed by the Hospital Universitari i Politècnic La Fe is as follows: The MicroSelectron afterloader together with the MicroSelectron‐v2 HDR ^192^Ir source was used in all measurements (Elekta AB, Stockholm, Sweden). The source was assayed using an electrometer CDX‐2000B and a well‐Type ionization chamber HDR 1000 Plus (Standard Imaging Inc. USA) calibrated in the Accredited Dosimetry Calibration Laboratory of the University of Wisconsin—Madison. The calibration was performed with the specific HDR ^192^Ir insert.

The Hospital Universitari i Politècnic La Fe has two calibrated SourceCheck 4π well chambers and electrometers available separately from the setup used in this work. These are calibrated every 2 years at the PTW Calibration Laboratory. These three measurement systems are cross‐calibrated regularly, at least once a year. Additionally, cross‐calibrations will also be considered if there was any suspicion regarding a measurement or if a value was observed outside established tolerances. This has not been the case for the well chamber and electrometer used in the present study, which have shown remarkable robustness during the period covered in this work. Additionally, the initial calibration coefficient has remained unmodified during the timeframe analyzed in this work.

Measurements are carried out following the installation of the source and prior to its use with patients. The response profile of the well chamber has been previously determined, so the measurement can be performed with the source at the center of the plateau. The measurement is performed by placing the chamber on a series of low‐density material pieces (polystyrene) to keep it away from the floor and walls, thus avoiding unwanted scattered radiation. The relative humidity has been consistently controlled, remaining within the correct operating range for the well chamber. The well chamber is stored in a cabinet inside the HDR bunker, ensuring environmental stability. In all cases, a proper warm‐up is considered to ensure the electrometer stability and any possible leakage currents are evaluated both pre‐ and post‐irradiation. The reading is then corrected for pressure and temperature conditions and then compared with the vendor's calibration certificate.

Following such procedure, we analyze measurements taken from March 2012 to January 2023. From this dataset, two different analyses are performed:

- The *S_K_* given by the vendor $\left(S_{K,vendor}\right)$ in the corresponding calibration certificate was compared with the value obtained in‐house $\left(S_{K,hosp}\right)$. *S_K,hosp_* and *S_K,vendor_* has to differ less than 3%.^5^ This ensures accurate BT deliver fulfilling current international recommendations.
- The *S_K_* of the source to be replaced $\left(S_{K,hosp}^{replace}\right)$ was measured and compared with the *S_K_* value given by the vendor $\left(S_{K,vendor}^{replace}\right)$, both adjusted by decay time. These values are then compared with the ones obtained at the time the source was installed $\left(S_{K,hosp}^{new}\mathrm{and}S_{K,vendor}^{new}\right)$. This method guarantees the long‐term stability of all the equipment involved in the measurements. It is also important to emphasize that this allows the clinic to record a detailed log of historical differences, allowing the clinical users the identification and correction of potential systematic deviations over time, even in those cases where a given medical physicist and/or equipment is no longer available. Since the measurement setup has to be performed in order to assay the new source, adding this additional measurement has no relevant impact on the clinical workflow.

To estimate the typical uncertainties for each value, a set of five measurements were performed. To do so, the well‐chamber was set and the insert and transfer tube were repositioned and reconnected before each measure.

## 3 RESULTS AND DISCUSSION

**FIGURE 1:**
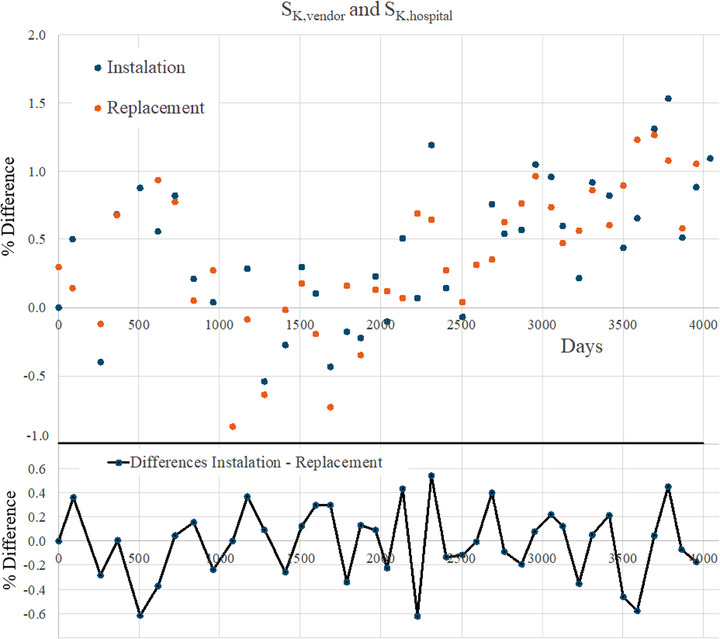
(Top panel) Difference (%) between *S_K, vendor_* and *S_K, hosp,_* (SK,hosp/SK,hospSK,vendorSK,vendor−1), as a function of the measurement date when the source is installed (blue dots) and replaced (orange dots). Day 0 corresponds to December 5, 2011. Error bars of 0.1% (*k = 2*) are not included for the sake of clarity. (Bottom panel) Difference (%) between *S_K, vendor_* and *S_K, hosp_* values at the time of replacement as compared to the time when the source was installed, (SK,hospreplace/SK,hospreplaceSK,vendorreplaceSK,vendorreplace−SK,hospnew/SK,hospnewSK,vendornewSK,vendornew). Lines are included to help visualization.

Percentage values for the difference between *S_K,vendor_* and *S_K,hosp_* (SK,hosp/SK,hospSK,vendorSK,vendor−1) are given in Figure 1 (top panel) as a function of the measurement date when the source is installed (blue dots) and replaced (orange dots). A standard deviation of 0.05% (*k = 2*) for individual measurement was observed, leading to typical uncertainties of less than 0.1% (*k = 2*) for such differences. All measurements are well within the recommended ±3% tolerance level, being the maximum deviation +1.5%. The existence of a statistically significant drift in this and similar datasets from other centers is currently being investigated by GEC‐ESTRO BRAPHYQS. Differences between *S_K,vendor_* and *S_K,hosp_* values at the time of replacement as compared to the time when the source was installed (SK,hospreplace/SK,hospreplaceSK,vendorreplaceSK,vendorreplace−SK,hospnew/SK,hospnewSK,vendornewSK,vendornew) are given in Figure 1 (bottom panel). In this case, differences are within the ±0.5% range, oscillating around a null systematic deviation. All measurements have been performed using the same equipment (well chamber, electrometer, and afterloader) in the same radiotherapy department using the procedure outlined above. Thus, emphasizing the stability and robustness of the results obtained. Considering that these are the maximum deviations reported during a 10 years period it is reasonable to define a ±0.5% tolerance for this component of the redundancy program. In the case of larger differences, the use of a different measurement set (well‐chamber, source‐holder insert, electrometer, barometer, and thermometer) is recommended. If the discrepancy persists, communication with the afterloader manufacturer is advised to clarify the disparity.

Recent GEC‐ESTRO ACROP recommendations^5^ clearly advocate for ensuring the constancy of all measurement equipment through an adequate redundancy program in such a way that the institution can guarantee that the dosimetry system has not changed over time. There, five different potential components of a redundancy program were suggested, being recommended that at least two of them have to be incorporated in the clinical workflow. The particular procedure analyzed in this work is among them, that is, “V. The decayed clinical source prior to exchange. This could be measured and the *S_K_* is compared (with decay correction) to the value originally measured upon source delivery to the clinic”. This solution has the advantage that measurements are performed on the same source at two different times.

The GEC‐ESTRO ACROP recommendations suggest that all components of the redundant system should be inter‐compared at least annually. However, the protocol described here enables more frequent assessments, occurring every 3−4 months during source exchange, thereby facilitating faster detection of any issues compared to the standard annual cross‐calibration. Although not explored here, this method can be directly applied to other radionuclides, for instance ^60^Co, where the source is measured at the time of installation and at least annually until replaced.

## 4 CONCLUSIONS

A straightforward quality assurance protocol with minimal impact on clinical workload has been outlined. This method entails an additional measurement when the BT source is replaced, ensuring a very simple, practical and efficient redundancy component to guarantee the long‐term stability of all equipment involved in the measurement. Additionally, it enables the hospital to maintain a detailed log of historical differences, facilitating the identification and correction of potential systematic deviations over time.

## AUTHOR CONTRIBUTIONS

The cited authors have contributed to this work and all of them agree with the text of this manuscript.

## CONFLICT OF INTEREST STATEMENT

The authors declare no conflicts of interest.
